# HDAC2 and HDAC5 Up-Regulations Modulate Survivin and miR-125a-5p Expressions and Promote Hormone Therapy Resistance in Estrogen Receptor Positive Breast Cancer Cells

**DOI:** 10.3389/fphar.2017.00902

**Published:** 2017-12-13

**Authors:** Wen-Tsung Huang, Yu-Hsuan Tsai, Shang-Hung Chen, Ching-Wen Kuo, Yao-Lung Kuo, Kuo-Ting Lee, Wen-Chung Chen, Pei Chih Wu, Chun-Yu Chuang, Siao Muk Cheng, Chun-Hui Lin, Euphemia Yee Leung, Yung-Chieh Chang, Chun Hei Antonio Cheung

**Affiliations:** ^1^Division of Hematology and Oncology, Department of Internal Medicine, Chi-Mei Medical Center, Liouying, Tainan, Taiwan; ^2^Department of Pharmacology, College of Medicine, National Cheng Kung University, Tainan, Taiwan; ^3^National Institute of Cancer Research, National Health Research Institutes, Tainan, Taiwan; ^4^Division of Oncology and Hematology, College of Medicine, National Cheng Kung University, Tainan, Taiwan; ^5^Department of Surgery, National Cheng Kung University Hospital, College of Medicine, National Cheng Kung University, Tainan, Taiwan; ^6^Department of Pathology, National Cheng Kung University Hospital, College of Medicine, National Cheng Kung University, Tainan, Taiwan; ^7^Department of Biomedical Engineering and Environmental Sciences, National Tsing Hua University, Hsinchu, Taiwan; ^8^Institute of Basic Medical Sciences, College of Medicine, National Cheng Kung University, Tainan, Taiwan; ^9^Auckland Cancer Society Research Centre and Department of Molecular Medicine and Pathology, University of Auckland, Auckland, New Zealand

## Abstract

Intrinsic or acquired resistance to hormone therapy is frequently reported in estrogen receptor positive (ER^+^) breast cancer patients. Even though dysregulations of histone deacetylases (HDACs) are known to promote cancer cells survival, the role of different HDACs in the induction of hormone therapy resistance in ER^+^ breast cancer remains unclear. Survivin is a well-known pro-tumor survival molecule and miR-125a-5p is a recently discovered tumor suppressor. In this study, we found that ER^+^, hormone-independent, tamoxifen-resistant MCF7-TamC3 cells exhibit increased expression of HDAC2, HDAC5, and survivin, but show decreased expression of miR-125a-5p, as compared to the parental tamoxifen-sensitive MCF7 breast cancer cells. Molecular down-regulations of HDAC2, HDAC5, and survivin, and ectopic over-expression of miR-125a-5p, increased the sensitivity of MCF7-TamC3 cells to estrogen deprivation and restored the sensitivity to tamoxifen. The same treatments also further increased the sensitivity to estrogen-deprivation in the ER^+^ hormone-dependent ZR-75-1 breast cancer cells *in vitro*. Kaplan–Meier analysis and receiver operating characteristic curve analysis of expression cohorts of breast tumor showed that high HDAC2 and survivin, and low miR-125a-5p, expression levels correlate with poor relapse-free survival in endocrine therapy and tamoxifen-treated ER^+^ breast cancer patients. Further molecular analysis revealed that HDAC2 and HDAC5 positively modulates the expression of survivin, and negatively regulates the expression miR-125a-5p, in ER^+^ MCF7, MCF7-TamC3, and ZR-75-1 breast cancer cells. These findings indicate that dysregulations of HDAC2 and HDAC5 promote the development of hormone independency and tamoxifen resistance in ERC breast cancer cells in part through expression regulation of survivin and miR-125a-5p.

## Introduction

Breast cancer is the most common type of cancer among women in both developed and developing countries. Typically, hormone therapy (e.g., selective ER modulators such as tamoxifen or aromatase inhibitors such as letrozole and anastrozole) is used to treat patients with ER^+^ breast cancer. Although ER^+^ breast cancer patients usually show good initial clinical response to hormone therapy, resistance to such treatment is frequently reported and the molecular mechanism underlying the induction of hormone therapy resistance in ER^+^ breast cancer is still incompletely understood (Holm et al., 2006).

Aberrant epigenetic alterations such as DNA hyper-methylation and histone hypo-acetylation can lead to chromatin remodeling, resulting in the down-regulation of various tumor suppressing genes like p53 and tazarotene-induced gene-1 (Tig1) (Takai et al., 2005). Histone acetyltransferases (HATs) and HDACs are enzymes that regulate the acetylation status of different histones in cells and accumulating evidence has revealed that dysregulation of certain HDAC isoforms can promote tumorigenesis, tumor metastasis, and drug-resistance induction. For examples, over-expression of HDAC1, HDAC4, and HDAC6 has recently been shown to promote the development of docetaxel, cisplatin, and temozolomide resistance in lung adenocarcinoma, ovarian, and glioblastoma cells, respectively (Stronach et al., 2011; Chen et al., 2014; Wang et al., 2016). In contrast, the role of HDAC2 and HDAC5 in the development of hormone therapy resistance in ER^+^ breast cancer has not yet been studied in details.

It is known that the ER^+^ human breast cancer cell line, MCF7, consists of highly heterogeneous breast cancer cells having significant genetic and phenotypic variations (e.g., differential tamoxifen and letrozole sensitivities) and MCF7-dervided, tamoxifen-resistant (or hormone-independent) sub-lines are widely used as models to study the induction of hormone therapy resistance in ER^+^ breast cancer (Planas-Silva et al., 2006; Huber-Keener et al., 2012; Zhou et al., 2012). In this study, we found that the MCF7-derived, ER^+^, estrogen-independent, tamoxifen-resistant MCF7-TamC3 breast cancer cells exhibit increased expression of HDAC2 and HDAC5 as compare to the estrogen-dependent, tamoxifen-sensitive MCF7 cells. Further molecular analysis revealed that the overexpressed HDAC2 and HDAC5 promote the development of hormone therapy resistance in ER^+^ breast cancer cells through multiple mechanisms including up-regulation of the pro-survival mTOR-survivin signaling pathway, and down-regulation of the tumor suppressing molecules, p53 and miR-125a-5p. Importantly, retrospective Kaplan–Meier analysis and ROC analysis showed that high HDAC2 and survivin, and low miR-125a-5p, expression levels significantly correlate with poor overall or relapse-free survival in tamoxifen or endocrine therapy-treated ER^+^ breast cancer patients. These findings indicate that dysregulations of HDAC2 and HDAC5 promote the development of hormone independency and tamoxifen resistance in ER^+^ breast cancer cells in part through expression regulations of survivin and miR-125a-5p.

## Materials and Methods

### Cell Lines and Cell Culture Conditions

Human breast adenocarcinoma MCF7 cells were cultured in α-MEM containing 5% FBS, PSG, and insulin-transferring-selenium supplement (ITS) (Roche, cat# 11074547001). The cellular and molecular phenotypes of the ER^+^ estrogen-independent and tamoxifen-resistant MCF7-TamC3 (**Supplementary Figure S1A**) have already been characterized in previous studies (Leung et al., 2010; Cheng et al., 2015). In brief, MCF7-TamC3 cancer cells were created by prolonged culture of the ER^+^ MCF7 cells (**Supplementary Figure S1A**) under estrogen-deprived conditions, which mimics the clinical effects of either oophorectomy or treatment with aromatase inhibitors such as letrozole (Jänicke, 2009; Leung and Baguley, 2013). MCF7-TamC3 cells were cultured in phenol-red-free RPMI containing 5% charcoal-stripped FBS, PSG, and ITS. The ER^+^ estrogen-dependent human breast carcinoma ZR-75-1 cells (**Supplementary Figure S1A**) were cultured in RPMI containing 10% FBS and PSG. All cells were incubated at 37°C under humidified atmosphere containing 5% CO_2_.

### Gene Silencing by siRNA

Target-validated siRNA oligomers were transfected into breast cancer cells using Lipofectamine^®^ RNAiMAX reagent (Thermo Fisher Scientific, cat# 13778150). The following siRNA oligomers were used in the study: survivin siRNA (Cell Signaling Technology, cat# 6351S); HDAC2 siRNA (Dharmacon, cat# M-003495-02); HDAC5 siRNA (Dharmacon, cat# M-003498-02); scramble siRNA (Dharmacon, cat# D-001206-13-05). Briefly, appropriate target-specific siRNA oligomers were diluted in Opti-MEM^®^ I medium (Thermo Fisher Scientific, cat# 11058021) without serum, and then mixed with Lipofectamine RNAiMAX^®^ transfection reagent diluted in Opti-MEM^®^ I medium without serum for 20 min at room temperature. Cells were overlaid with the transfection mixture and incubated for various durations.

### 3-(4,5-Dimethylthiazol-2-yl)-2,5-Diphenyltetrazolium Bromide (MTT) Cell Viability Assay

A total of 5,040 cells were seeded onto each well of 96-well plates a day prior to various treatments. After treatment, 180 μL MTT solutions (mixing MTT 5 mg/mL in phenol-red free RPMI in a ratio of 1:10) was added to each well and incubated for 4 h. Then, 100 μL MTT lysis buffer was added to each well and incubated for 16 h. The absorbance of the solution was quantified by measuring at 570 nm wavelength by a spectrophotometer. The percentage of viable cells for each treatment group was calculated by adjusting the untreated control group to 100%. Duplicate wells were assayed for each condition.

### Lactate Dehydrogenase (LDH) Cell Cytotoxicity Assay

Cell cytotoxicity assay was performed using the LDH-cytotoxicity assay kit II (Abcam, cat# ab65393). Briefly, cells were seeded at 5,040 cells/well in 96-well plates for 24 h prior to the treatments. Cell cytotoxicity was quantified by measuring the absorbance of the solution at a 450 nm wavelength using a SpectraMax M5 microplate reader (Molecular Devices LLC, United States). Cytotoxicity index for each treatment group was calculated using the equation: (Test sample – Low control)/(High control – Low control), and also by adjusting the untreated control group to 1. Test sample – cells transfected with scramble siRNA only, cells with transfected HDAC2 siRNA, or cells transfected with survivin siRNA; low control – completely untreated cells (minimal LDH-value); high control – completely lysed cells (maximal LDH-value).

### Western Blot Analysis

Cells were lysed using the CelLytic^TM^ cell lysis reagent (Sigma–Aldrich, cat# C2978) that contained 1 mM PMSF, 1 mM NaF, cocktail protease inhibitors (Roche, cat# 04693159001), and phosphatase inhibitors (G-Biosciences, cat# 1786-450). Equal amounts of protein were subjected to SDS-PAGE on a 6%, 8%, or 10% polyacrylamide gel. The resolved proteins were transferred onto a PVDF membrane (Merck Millipore, cat# IPVH00010), which was then exposed to 5% non-fat-dried milk or 3% bovine serum albumin in Tris-buffered saline containing 0.1% Tween 20 (TBST) for an hour at room temperature before incubation overnight at 4°C with different primary antibodies: anti-survivin (Cell Signaling Technology, cat# 71G4B7); anti-p-survivin (Abcam, cat# ab10720); anti-HDAC2 (Genetex, cat# GTX109642); anti-HDAC5 (Protein Tech, cat# 161661-AP); anti-Atg5 (Millipore, cat# MAB2605); anti-LC3B (Origene, cat# TA301543); anti-p53 (Genetex, cat# GTX102965); anti-Sp1 (Millipore, cat# 07-645); anti-p62/SQSTM1 (Genetex, cat# GTX100685); anti-Bcl-2 (Genetex, cat# GTX100064); anti-HER2 antibody (UltraMAB, cat# UM570036); anti-p-mTOR (Cell Signaling Technology, cat# 2971); anti-mTOR (Cell Signaling Technology, cat# 2972S); anti-p-Akt (Cell Signaling Technology, cat# 2965); anti-Akt (Cell Signaling Technology, cat# 9272S); anti-actin (Millipore, cat# MAB1501). The PVDF membrane was then washed with TBS containing 0.05% Tween-20 before incubation for an hour at room temperature with different horse-radish peroxidase–conjugated secondary antibodies. Immune complexes were finally detected with chemiluminescence reagents, and luminescence protein signals were detected by Luminescence Readers (FUJI LAS-100, Fujifilm, Japan).

### RNA Extraction and Quantitative Reverse Transcriptase-Polymerase Chain Reaction (qRT-PCR) Analysis

Total RNA was extracted using TRIzol^®^ reagent (Thermo Fisher Scientific, cat# 15596-026) and complementary DNA (cDNA) was synthesized from RNA using the RevertAid H Minus First strand cDNA synthesis kit (Thermo Fisher Scientific, cat# K1631). qRT-PCR was used to determine the relative expression levels of survivin, HDAC2, and HDAC5 in cells by using the StepOnePlus^TM^ Real-Time PCR System (Thermo Fisher Scientific, United States). The specific primers with the following sequences were used in the study: human survivin forward primer, 5′-CTGCCTGGCAGCCCTTT-3′; survivin reverse primer, 5′-CCTCCAAGAAGGGCCAGTTC-3′; human actin forward primer, 5′-GGCGGCACCACCATGTACCCT-3′; human actin reverse primer, 5′-AGGGGCCGGACTCGTCATACT-3′; human HDAC2 forward primer, 5′-GCTATTCCAGAAGATGCTGTT-3′; human HDAC2 reverse primer, 5′-TCGACCTCCTTCTCCTTCATCC-3′; human HDAC5 forward primer, 5′-CGCAAGGATGGGACTGTTAT-3′; human HDAC5 reverse primer, 5′-GAGCATCTCAGTGGGGATGT-3′. A TaqMan microRNA assay (ID 002198 – has-miR-125a-5p; ID 001093 – RNU6B) was used to determine the expression of miR-125a-5p in MCF7, MCF7-TamC3, and ZR-75-1 cells.

### Immunofluorescent Microscopy

MCF7 and MCF7-TamC3 cells were seeded on glass coverslips for 48 h. Cells were then fixed with 4% paraformaldehyde at room temperature for 15 min, washed three times with ice cold PBS, permeabilized with PBST (PBS containing 1% triton X-100) for 30 min, and blocked in solution containing 5% bovine serum albumin (Sigma–Aldrich, cat# A2153) for an hour at room temperature. The cells were incubated with primary antibody [anti-HER2 antibody (UltraMAB, cat# UM570036)] at 4°C overnight and washed three times with TBST, followed by incubation with secondary antibody for an hour at room temperature. Cells were washed three times with TBST and the slides were mounted with glycerol-gelatin (Sigma–Aldrich). Nuclei were counterstained by DAPI. The images were taken by scanning confocal microscope (MPE, Olympus). The localization of different proteins in confocal images was pixel-by-pixel analyzed by FV-1000 software.

### Kaplan–Meier Survival Analysis and Receiver Operating Characteristic Curve (ROC) Analysis

The overall survival and relapse-free survival of patients with ER^+^ tamoxifen/endocrine therapy-treated breast cancer stratified by HDAC2, HDAC3, HDAC5, or survivin (*BIRC5*) expression levels (low and high) were evaluated using Kaplan–Meier analysis from a large publicly available clinical breast cancer microarray online database and web tool (Kaplan Meier plotter^1^) (Györffy et al., 2010; Gyórffy et al., 2014). The overall survival of patients with ER^+^ tamoxifen-treated breast cancer stratified by miR-125a-5p expression levels (low and high) were evaluated using Kaplan–Meier analysis from a publicly available prognostic miRNA online database and web tool (PROGmiR V2^2^) (Goswami and Nakshatri, 2012). The ROC analysis was constructed to quantify the accuracy of target genes (*HDAC2* and *BIRC5*) using the SigmaPlot SPW10.0 software. The area under the curve (AUC) is a combined measure of sensitivity and specificity between 0 and 1. A test with an AUC value of 1 means perfect accuracy. The Sp1/miR-125a-5p interaction was predicted using miRNA target prediction software (TargetScanHuman 7^3^ and PicTar^4^).

### Statistical Analysis

Each experiment was performed at least three times. Data are presented as mean ± SEM. The significance of difference was evaluated with one-way analysis of variance (one-way ANOVA). A *p*-value < 0.05 was considered statistically significant.

## Results

### Estrogen-Independent MCF7-TamC3 Cells Exhibit Increased Expression of HDAC2 and HDAC5 As Compared to the Parental MCF7 Cells

**FIGURE 1:**
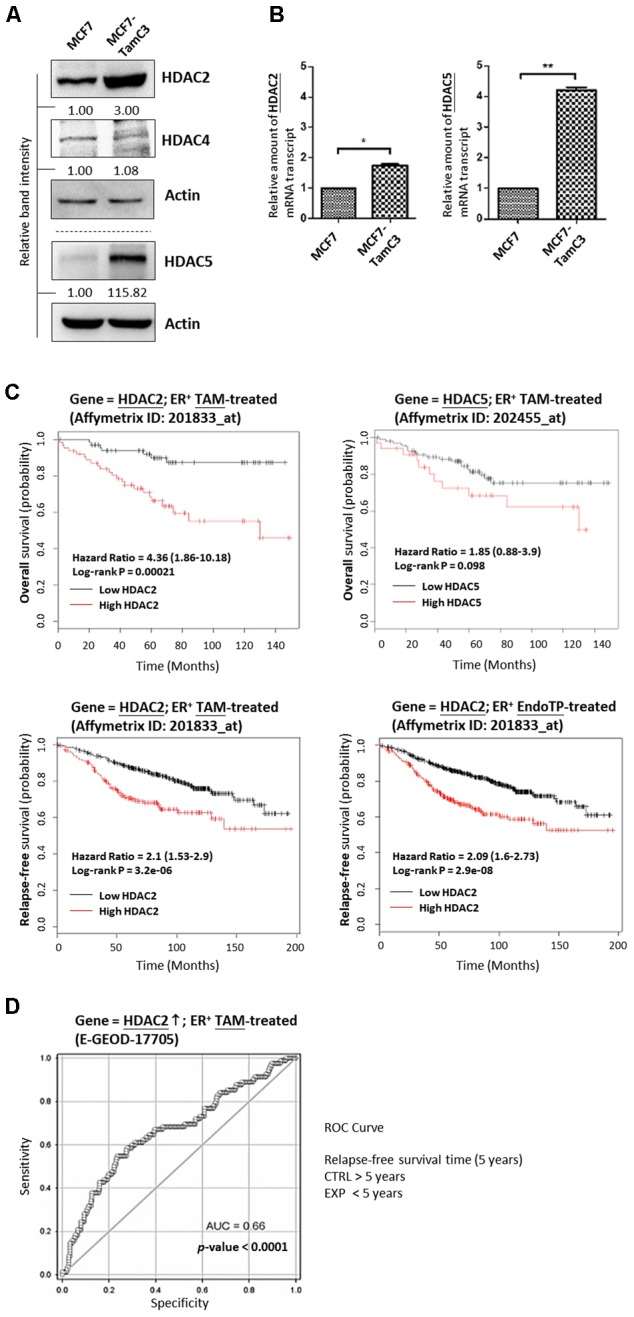
Upregulation of HDAC2 correlates with poor overall and relapse-free survival in patients with ER^+^, hormone therapy-treated breast cancer. **(A,B)** Expression of various HDAC isoforms in MCF7 and MCF7-TamC3 cells was determined by Western blotting and qPCR analysis. Both “^∗^” and “^∗∗^” denote a statistical significance (*P* < 0.05 and *P* < 0.01, respectively) between the testing groups. **(C)** Kaplan–Meier survival estimates of high (red line) or low (black line) HDAC2 and HDAC5 expression in ER^+^ tamoxifen/endocrine therapy-treated breast cancer. Analysis was performed using the online database and web tool (Kaplan Meier plotter). **(D)** ROC analysis of HDAC2 for 5-year relapse-free survival on ER^+^ tamoxifen-treatment breast cancer patients.

An MCF7-derived, estrogen-independent and tamoxifen-resistant breast cancer cell line, MCF7-TamC3, was used in this study. Western blot and qPCR analysis revealed that the expression of HDAC2 and HDAC5, but not of HDAC4, is significantly increased in MCF7-TamC3 cells, as compared to the parental estrogen-dependent tamoxifen-sensitive MCF7 cells (**Figures 1A,B**). At the clinical level, Kaplan–Meier analysis of expression cohorts of breast tumor showed that high HDAC2 expression levels significantly (*p*-value < 0.001) correlate with poor relapse-free survival and poor overall survival [hazard ratio (HR) > 2] in tamoxifen or endocrine therapy-treated ER^+^ breast cancer patients (**Figure 1C**). In addition, ROC analysis for 5-year relapse-free survival on ER^+^ tamoxifen-treatment breast cancer patients showed an AUC of 0.66 (95% CI: 0.59–0.74; *p*-value < 0.0001) (**Figure 1D**). Despite not reaching statistical significance, high HDAC5 expression levels also correlate with poor overall survival (HR = 1.85) in tamoxifen-treated ER^+^ breast cancer patients (**Figure 1C**). Collectively, these results suggest that aberrant expression of HDAC2 and HDAC5 may affect the effectiveness of hormone therapy in patients with ER^+^ breast cancer.

### Down-Regulation of HDAC2 and HDAC5 Partially Restores the Sensitivity to Tamoxifen and Increases the Sensitivity to Estrogen-Deprivation in MCF7-TamC3 Cells

**FIGURE 2:**
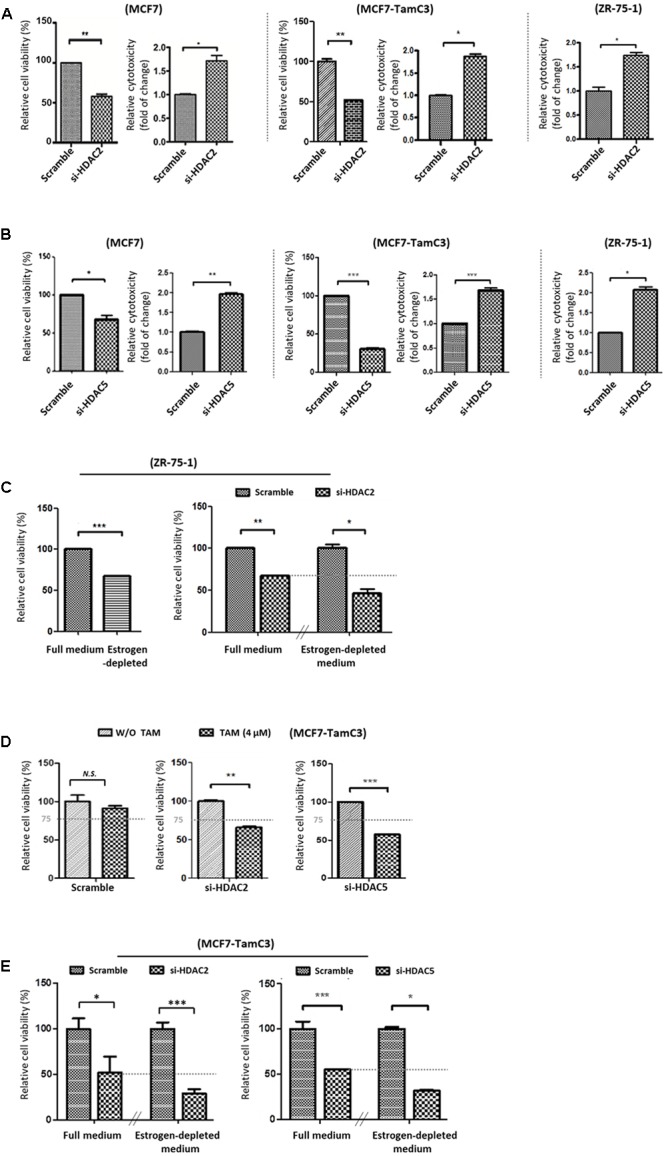
Downregulation of HDAC2 and HDAC5 increases the sensitivity to tamoxifen and restores the sensitivity to estrogen deprivation in MCF7-TamC3 cells. **(A,B)** MCF7, MCF7-TamC3, and ZR-75-1 cells were transfected with scramble siRNA, HDAC2 siRNA, or HDAC5 siRNA. Cell viability and cytotoxicity was assessed by the MTT assay (96 h post-treatment) and LDH assay (5 days post-treatment), respectively. **(C)** Left panel: ZR-75-1 cells were cultured under either estrogen-containing (full medium) or estrogen-deprived conditions for 96 h. Cell viability was assessed by the MTT assay. Right panel: ZR-75-1 cells were pre-transfected with either scramble siRNA or HDAC2 siRNA for 24 h and subsequently cultured under either estrogen-containing or estrogen-deprived conditions for 96 h. Cell viability was assessed by the MTT assay. **(D)** MCF7-TamC3 cells were pre-transfected with scramble siRNA, HDAC2 siRNA, or HDAC5 siRNA for 24 h and co-treated with or without tamoxifen for 72 h. Cell viability was assessed by the MTT assay. **(E)** MCF7-TamC3 cells were pre-transfected with scramble siRNA, HDAC2 siRNA, or HDAC5 siRNA for 24 h and subsequently cultured under either estrogen-containing or estrogen-deprived conditions for 72 h. Cell viability was assessed by the MTT assay. “^∗^”^,^ “^∗∗^”^,^ and “^∗∗∗^” denote a statistical significance (*P* < 0.05, *P* < 0.01, and *P* < 0.001, respectively) between the testing groups. “*N.S.*” denotes no statistical significance between the testing groups.

We next examined the role of HDAC2 and HDAC5 in the survival of ER^+^ breast cancer cells. Molecular down-regulation of HDAC2 and HDAC5 by siRNA decreased the cell viability of MCF7 and MCF7-TamC3. HDAC2 siRNA and HDAC5 siRNA also promoted the death of MCF7, MCF7-TamC3, and the ER^+^ tamoxifen-sensitive ZR-75-1 breast cancer cells (Cameron et al., 1997) (**Figures 2A,B**). Down-regulation of HDAC2 by siRNA further decreased the viability of ZR-75-1 cells cultured under estrogen-deprived conditions (i.e., reduced by 52% in estrogen-deprived medium vs. 29% in full medium), suggesting HDAC2 may exhibit an enhanced pro-cell survival role in ER^+^ breast cancer cells experiencing estrogen-deprived stress (**Figure 2C**).

We subsequently investigate the role of HDAC2 and HDAC5 in promoting the induction of hormone therapy resistance in MCF-TamC3 cells. Cell viability analysis revealed that down-regulation of HDAC2 and HDAC5 restored the sensitivity to tamoxifen (4 μM; IC_50_ of MCF7 and ∼1/4 IC_50_ of MCF7-TamC3) in MCF7-TamC3 cells under the estrogen-containing conditions (**Figure 2D**). Down-regulation of HDAC2 and HDAC5 also further decreased the viability of MCF7-TamC3 cells cultured under estrogen-deprived conditions, indicating that the over-expressed HDAC2 and HDAC5 in part contributes to the decreased sensitivity to tamoxifen and the increased tolerability to estrogen deprivation in MCF7-TamC3 cells (**Figure 2E**).

### MCF7-TamC3 Cells Exhibit Increased Activation of the Pro-Survival mTOR-Survivin Signaling Pathway

**FIGURE 3:**
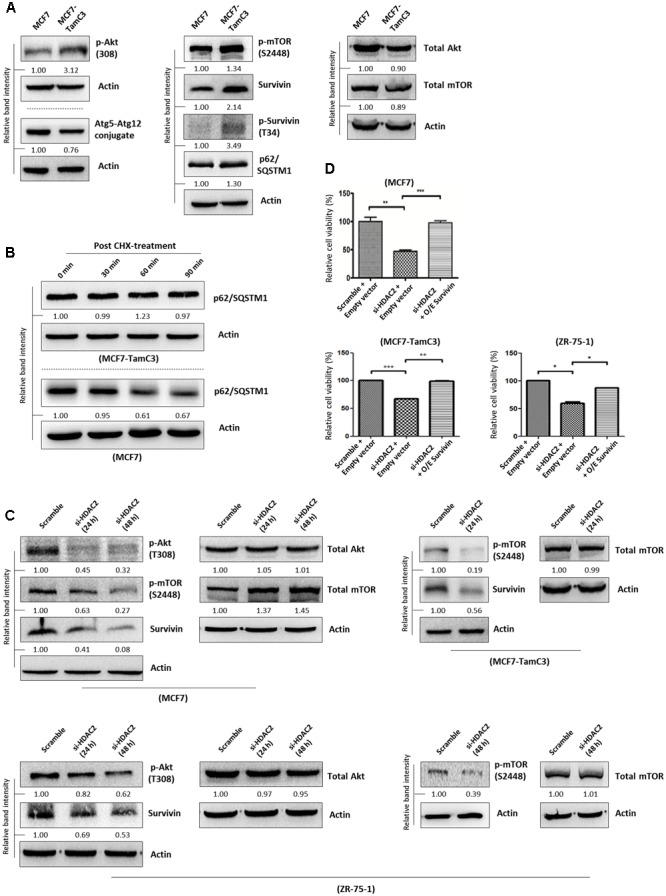
HDAC2 positively regulates the expression of p-Akt, p-mTOR, and survivin in ER^+^ breast cancer cells. **(A)** Expression of different proteins in MCF7 and MCF7-TamC3 cells was determined by Western blotting. **(B)** MCF7 and MCF7-TamC3 cells were treated with 10 μg/mL cycloheximide (CHX) for 30 min to inhibit *de novo* protein synthesis. The expression of p62/SQSTM1 30 min, 60 min, and 90 min post-CHX treatment was determined by Western blotting. **(C)** Breast cancer cells were transfected with either scramble siRNA or HDAC2 siRNA for 24–48 h and expression of different proteins was determined by Western blotting. **(D)** MCF7, MCF7-TamC3, and ZR-75-1 cells were pre-transfected with either pCMV6-XL4 (empty plasmid) or pCMV6-XL4-survivin (O/E survivin) for 24 h and subsequently treated with or without HDAC2 siRNA for 96 h. Cell viability was assessed by the MTT assay. “^∗^”^,^ “^∗∗^”^,^ and “^∗∗∗^” denote a statistical significance (*P* < 0.05, *P* < 0.01, and *P* < 0.001, respectively) between the testing groups.

A previous study showed that myocardium isolated from the HDAC2-null mice exhibited reduced expression of p-Akt and p-mTOR as compared to the HDAC2-wild-type mice (Trivedi et al., 2007). Therefore, we speculated that the increased expression of HDAC2 might lead to the up-regulation of the pro-survival Akt-mTOR-survivin pathway in MCF7-TamC3 cells. Here, results of the Western blot analysis showed that pharmacological inhibition of mTOR by rapamycin decreased the expression of survivin and p62/SQSTM1 (autophagic flux indicator) in MCF7, MCF7-TamC3, and ZR-75-1 cells, confirming that mTOR positively modulates survivin expression and negatively regulates autophagy in the tested ER^+^ breast cancer cells (**Supplementary Figure S2**). Interestingly, Western blot analysis revealed that MCF7-TamC3 cells overexpress p-Akt, p-mTOR, survivin and its active form p-survivin as compared to MCF7 cells (**Figure 3A**). Coinciding with the functions of mTOR and survivin as autophagy negative regulators (Cheng et al., 2015; Vequaud et al., 2015; Lee et al., 2016), the p-mTOR and survivin co-upregulated MCF7-TamC3 cells also exhibit decreased expression of Atg5-Atg12 conjugate (an indicator for autophagophore elongation reduction), increased expression of p62/SQSTM1 (an indicator for autophagic flux reduction), and increased p62/SQSTM1 protein stability (an indicator for autophagic flux reduction) as compared to the parental MCF7 cells (**Figures 3A,B**).

Next, we sought to determine whether HDAC2 plays a role in the up-regulation of the pro-survival mTOR-survivin pathway in MCF7-TamC3 cells. Down-regulation of HDAC2 by siRNA decreased the expression of p-Akt, p-mTOR, and survivin in MCF7, MCF7-TamC3, and ZR-75-1 cells (**Figure 3C**). Importantly, ectopic over-expression of survivin attenuated the cell viability reduction effect caused by HDAC2 siRNA in MCF7, MCF7-TamC3, and ZR-75-1 cells, confirming the pro-survival role of the HDAC2-mTOR-survivin signaling pathway in ER^+^ breast cancer cells (**Figure 3D**).

### Down-Regulation of Survivin Partially Restores the Sensitivity to Tamoxifen and Increases the Sensitivity to Estrogen-Deprivation in MCF7-TamC3 Cells

**FIGURE 4:**
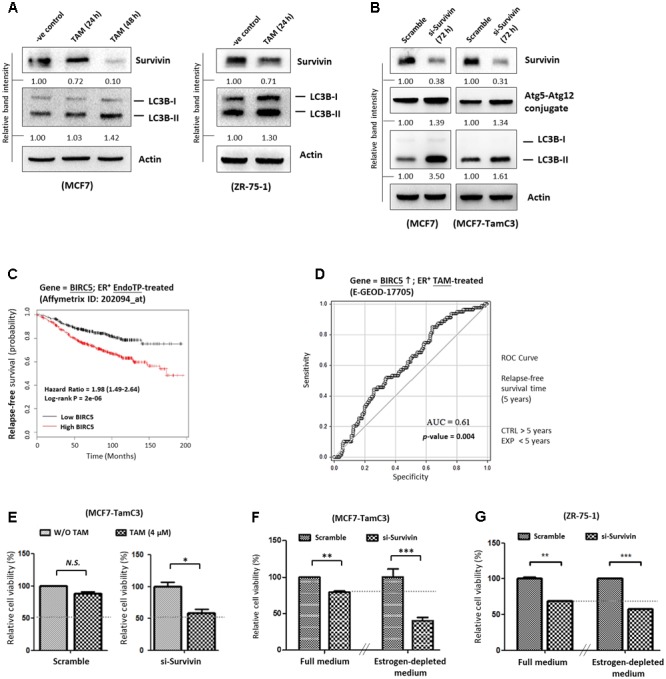
Overexpression of survivin contributes to the induction of hormone therapy resistance in the ER^+^ HDAC2-upregulated breast cancer cells. **(A)** MCF7 and ZR-75-1 cells were treated with 4 μM tamoxifen for 24–48 h and expression of survivin and conversion of LC3B-II were determined by Western blotting. **(B)** MCF7 and MCF7-TamC3 cells were transfected with either scramble siRNA or survivin siRNA for 72 h. Expression of survivin, Atg5-Atg12 conjugate, and conversion of LC3B-II were determined by Western blotting. Actin was used as an internal control. **(C)** Kaplan–Meier survival estimates of high (red line) or low (black line) survivin expression in ER^+^ endocrine therapy-treated breast cancer. **(D)** ROC analysis of BIRC5 (survivin) for 5-year relapse-free survival on ER^+^ tamoxifen-treatment breast cancer patients. **(E)** MCF7-TamC3 cells were pre-transfected with either scramble siRNA or survivin siRNA for 24 h and co-treated with or without tamoxifen for 72 h. Cell viability was assessed by the MTT assay. **(F)** MCF7-TamC3 cells were pre-transfected with either scramble siRNA or survivin siRNA for 24 h and subsequently cultured under either estrogen-containing (Full) or estrogen-deprived conditions for 72 h. **(G)** ZR-75-1 cells were pre-transfected with either scramble siRNA or survivin siRNA for 24 h and subsequently cultured under either estrogen-containing (Full) or estrogen-deprived conditions for 72 h. Cell viability was assessed by the MTT assay. “^∗^”^,^ “^∗∗^”^,^ and “^∗∗∗^” denote a statistical significance (*P* < 0.05, *P* < 0.01, and *P* < 0.001, respectively) between the testing groups.

17β-estradiol-induced ER activation was shown to trigger survivin expression in ovarian cancer cells whereas targeting the ER signaling pathway by tamoxifen was shown to down-regulate survivin expression, leading to the induction of cell death in human hepatoblastoma and colorectal cancer cells (Guo et al., 2010; Morad et al., 2012; Zhu et al., 2012). Therefore, the effects of HDAC2-survivin up-regulation on the induction of hormone therapy-resistance were further investigated in MCF7-TamC3 cells. Here, Western blot analysis showed that tamoxifen decreased survivin expression and increased LC3B-II conversion in tamoxifen-sensitive MCF7 and ZR-75-1 cells as expected (**Figure 4A**). Similar to the results of MCF7 cells treated with tamoxifen, down-regulation of survivin by siRNA also increased LC3B-II conversion in MCF7 and MCF7-TamC3 cells (**Figure 4B**). At the clinical level, retrospective Kaplan–Meier analysis of expression cohorts of breast tumor showed that high survivin (*BIRC5*) expression levels significantly (*p*-value < 0.0001) correlate with poor relapse-free survival (HR = 1.98) in endocrine therapy-treated ER^+^ breast cancer patients (**Figure 4C**). In addition, ROC analysis for 5-year relapse-free survival on ER^+^ tamoxifen-treatment breast cancer patients showed an AUC of 0.61 (95% CI: 0.54–0.68; *p*-value = 0.004) (**Figure 4D**).

Further investigations were carried out to confirm the role of survivin in modulating the sensitivity to hormone therapy in ER^+^ breast cancer cells. As shown in **Figures 4E,F**, down-regulation of survivin by siRNA restored the sensitivity to tamoxifen and increased the sensitivity to estrogen-deprivation in MCF-TamC3 cells. Moreover, down-regulation of survivin by siRNA further decreased the viability of ZR-75-1 cells cultured under the estrogen-deprived conditions as compared the cells cultured under estrogen-containing medium (**Figure 4G**). These results support that up-regulation of the HDAC2-modulated survivin expression contributes to the induction of hormone therapy-resistance in MCF7-TamC3 cells.

### MCF7-TamC3 Cells Exhibit Increased Sp1 and Decreased p53 Expressions As Compared to MCF7 cells

**FIGURE 5:**
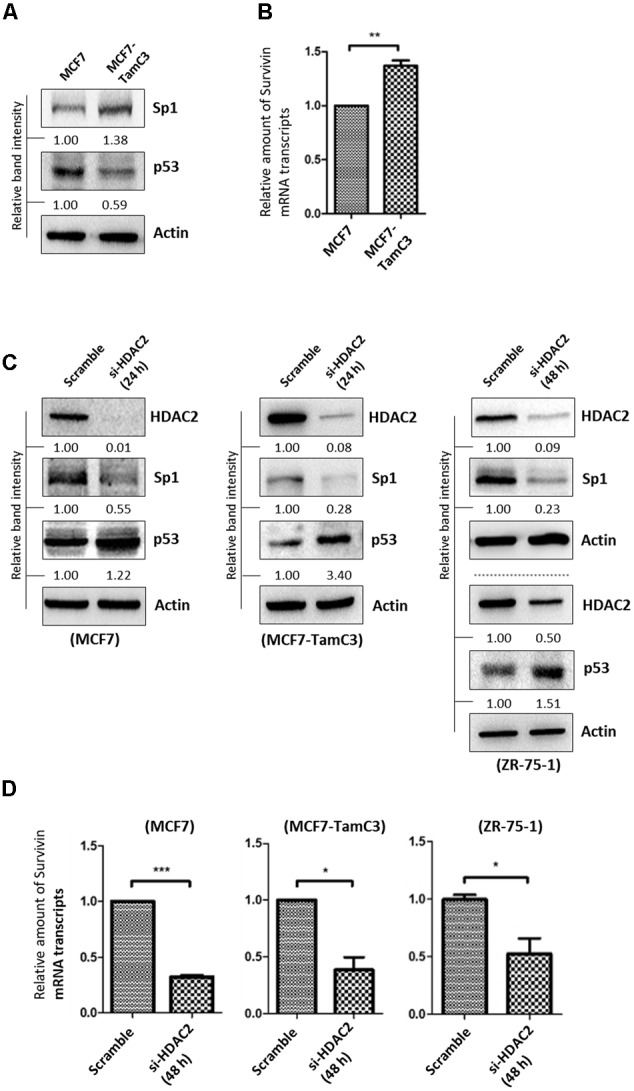
HDAC2 positively regulates the expression of Sp1 and negatively regulates the expression of p53 in ER^+^ breast cancer cells. **(A)** Expression of Sp1 and p53 in MCF7 and MCF7-TamC3 cells was determined by Western blotting. **(B)** Expression of survivin at the transcriptional level in MCF7 and MCF7-TamC3 cells was determined by qPCR. “^∗∗^” denotes a statistical significance (*P* < 0.01) between the testing groups. **(C)** Breast cancer cells were transfected with either scramble siRNA or HDAC2 siRNA for 24–48 h (depending on the target knockdown efficiency in different cell lines). Expression of Sp1 and p53 was determined by Western blotting. **(D)** Breast cancer cells were transfected with either scramble siRNA or HDAC2 siRNA for 48 h and expression of survivin was determined by qPCR. “^∗^” and ^∗∗∗^” denote a statistical significance (*P* < 0.05 and *P* < 0.001, respectively) between the testing groups.

The ER^+^ breast cancers (e.g., luminal A-subtype) are predominantly p53 wild-type (p53^WT^) and p53 is known to negatively regulate survivin gene transcription at the molecular level (Bailey et al., 2012; Berger et al., 2013; Dumay et al., 2013). In contrast, Sp1 positively regulates survivin gene transcription (Hoffman et al., 2002; Xu et al., 2007; Raj et al., 2008; Chen et al., 2011). Interestingly, network analysis (STRING ver.10) showed that HDAC2 and HDAC5 can interact with the transcription factor p53 and Sp1 in cells (**Supplementary Figure S3A**). Here, Western blot analysis revealed that the p53^WT^-expressing MCF7-TamC3 cells exhibit increased expression of Sp1 but decreased expression of p53 as compared to MCF7 cells (**Figure 5A**). As expected, MCF7-TamC3 cells exhibit increased expression of survivin at the transcriptional level (**Figure 5B**).

To investigate possible links between HDAC2, p53, Sp1, and survivin expression in ER^+^ breast cancer cells, expressions of p53 and Sp1 in ER^+^ breast cancer cells treated with HDAC2 siRNA were determined. Down-regulation of HDAC2 decreased Sp1 but increased p53 expressions in MCF7, MCF7-TamC3, and ZR-75-1 cells (**Figure 5C**). In agreement with the predicated effects of Sp1 down-regulation and p53 up-regulation on survivin expression at the transcriptional level, HDAC2 down-regulation decreased the amount of survivin mRNA transcripts present in MCF7, MCF7-TamC3, and ZR-75-1 cells (**Figure 5D**). Furthermore, inhibition of p53 by pifithrin-α partially attenuated the expression down-regulatory effect of HDAC2 siRNA on survivin in MCF7 cells (**Supplementary Figure S3B**). Collectively, these results indicate that HDAC2 over-expression up-regulates survivin expression in part through alterations of both Akt-mTOR (at the translational level) and Sp1/p53 (at the transcriptional level) signaling pathways.

### MCF7-TamC3 Cells Exhibit Decreased Expression of the Tumor Suppressor, miR-125a-5p, As Compared to MCF7 Cells

**FIGURE 6:**
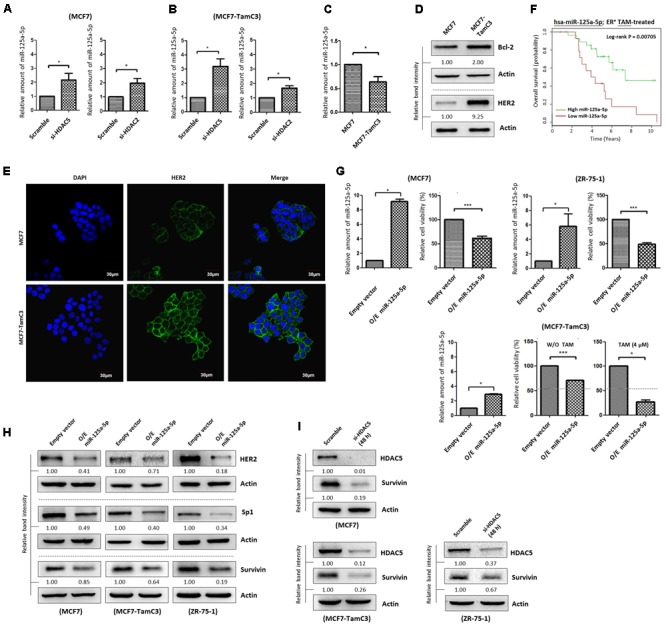
HDAC2 and HDAC5 negatively regulate the expression of miR-125a-5p in ER^+^ breast cancer cells. **(A,B)** Breast cancer cells were transfected with scramble siRNA, HDAC2 siRNA, or HDAC5 siRNA for 48 h and expression of miR-125a-5p was determined by qPCR. “^∗^” denotes a statistical significance (*P* < 0.05) between the testing groups. **(C)** Expression of miR-125a-5p in MCF7 and MCF7-TamC3 cells was determined by qPCR. **(D)** The expression of Bcl-2 and HER2 in MCF7 and MCF7-TamC3 cells were determined by Western blotting. **(E)** The cell surface expression HER2 (green) in MCF7 and MCF7-TamC3 cells was assessed by immunofluorescence confocal microscopy. Nuclei were countered stained by DAPI (blue). **(F)** Kaplan–Meier survival estimates of high (green line) or low (red line) miR-125a-5p expression in ER^+^ tamoxifen-treated breast cancer. **(G)** MCF7 and ZR-75-1 cells were transfected with either pLV-[mir-control] (empty) or pLV-[has-mir-125a-5p] (O/E miR-125a-5p) for 96 h. MCF7-TamC3 cells were pre-transfected with either pLV-[mir-control] or pLV-[has-mir-125a-5p] for 24 h and subsequently treated with or without tamoxifen for 72 h. Cell viability was determined by the MTT assay. “^∗^” and ^∗∗∗^” denote a statistical significance (*P* < 0.05 and *P* < 0.001, respectively) between the testing groups. **(H)** Breast cancer cells were transfected with either pLV-[mir-control] or pLV-[has-mir-125a-5p] for 48 h and expression of, HER2, Sp1, and survivin was determined by Western blotting. Actin was used as an internal control. **(I)** MCF7, MCF7-TamC3, and ZR-75-1 cells were transfected with either scramble or HDAC5 siRNA for 48 h and expression of survivin was determined by Western blotting.

Hsieh et al. (2015) showed that the expression of a newly discovered tumor suppressor, microRNA 125a-5p (miR-125a-5p), was induced by silencing of HDAC5 in the ER^+^/HER2^+^ R2N1d breast cancer cells in a dose-dependent manner. We therefore hypothesized that the over-expressed HDAC5 may promote miR-125a-5p down-regulation, leading to the enhanced cell survival in MCF7-TamC3 cells under estrogen deprivation. We confirmed that HDAC5 down-regulation increased miR-125a-5p expression in MCF7 and MCF7-TamC3 by qPCR analysis (**Figures 6A,B**). In addition, HDAC2 down-regulation also increased the expression of miR-125a-5p in the tested breast cancer cells (**Figures 6A,B**). In agreement with the negative regulatory functions of HDAC5 and HDAC2 on miR-124a-5p expression, MCF7-TamC3 cells exhibit decreased expression of the miR-125a-5p as compared to MCF7 cells (**Figure 6C**). Western blot analysis and confocal microscopic analysis revealed that the expression of the two known miR-125a-5p negatively regulating pro-breast cancer cell survival molecules, Bcl-2 and HER2, is also increased in MCF7-TamC3 cells as compared to MCF7 cells (**Figures 6D,E**) (Fassan et al., 2013; Tong et al., 2015). Of interest, retrospective Kaplan–Meier analysis of expression cohorts of breast tumor showed that low miR-125a-5p expression levels correlate with poor overall survival in tamoxifen-treated ER^+^ breast cancer patients (**Figure 6F**).

The role of miR-125a-5p in modulating the sensitivity to hormone therapy in ER^+^ breast cancer cells was further investigated *in vitro*. Ectopic over-expression of miR-125a-5p decreased the viability of MCF7, ZR-75-1, and MCF7-TamC3 cells, confirming the role of miR-125a-5p as a tumor suppressing molecule (**Figure 6G**). Importantly, ectopic over-expression of miR-125a-5p restored the sensitivity to tamoxifen (4 μM) in MCF7-TamC3 cells (**Figure 6G**). Interestingly, the miRNA target prediction online software, TargetScan and PicTar, showed that Sp1 harbors a miR-125a-5p seed sequence, and further molecular analysis revealed that ectopic over-expression of miR-125a-5p decreased the expression of both Sp1 and survivin in MCF7, MCF7-TamC3, and ZR-75-1 cells (**Supplementary Figure S4A** and **Figure 6H**). Because HDAC5 negatively regulates miR-125a-5p expression, we suspected that HDAC5 up-regulation might also in part contributes to the Sp1 and survivin over-expression found in MCF7-TamC3 cells. Here, down-regulation of HDAC5 by siRNA clearly decreased the expression of Sp1 (24 h post-treatment) and survivin (48 h post-treatment) in the tested ER^+^ breast cancer cells, indicating that HDAC5 positively regulates the expression of Sp1 and survivin, and suggesting that HDAC5 may promote the induction of hormone therapy resistance in MCF7-TamC3 cells, in part through alteration of the miR-125a-5p-Sp1-survivin signaling pathway (**Figure 6I** and **Supplementary Figure S4B**).

## Discussion

Breast cancer is the most common type of cancer among women in both developed and developing countries. Recently, it has been shown that high expression of HDAC2 correlates with poor prognosis in breast cancer patients receiving anthracyclines therapy and that HDAC2 negatively modulates the DNA binding activity of p53 in MCF7 cells. However, the molecular role/s of HDAC2 in regulating ER^+^ breast cancer cell survival and hormone therapy resistance induction is still largely unknown (Harms and Chen, 2007; Zhao et al., 2016). Here, we found that both HDAC2 and HDAC5 are up-regulated in the estrogen-independent tamoxifen-resistant MCF7-TamC3 cells. Importantly, we also found that MCF7-TamC3 cells (with HDAC2 and HDAC5 up-regulations) exhibit increased expression of various pro-survival molecules including survivin and mTOR, and decreased expression of different tumor suppressors like p53 and miR-125a-5p.

Aberrant regulations of the Akt-mTOR-survivin and the p53/Sp1-survivin signaling pathways have widely been shown to promote the survival of cancer cells and the induction of anti-cancer drugs resistance (Cheung et al., 2009; Coumar et al., 2013; Dong et al., 2014; Sun et al., 2014; Han et al., 2015; Parvani et al., 2015; Kim et al., 2016). It is not surprising to see that the HDAC2 up-regulated MCF7-TamC3 cells exhibit increased endogenous expression of survivin as compared to the parental hormone therapy-sensitive MCF7 cells because p53 is a negative transcription regulator of the survivin gene (Mirza et al., 2002). However, reduced p53 expression may also affect survivin expression at the translational level in MCF7-TamC3 cells. A previous study revealed that p53 negatively regulates the PI3K/Akt signaling pathway through up-regulated expression of IGF-BP3 and PTEN (Buckbinder et al., 1995). Moreover, p53 also negatively regulates mTOR activity through up-regulation of AMPK-β, Sestrins 1/2, TSC2, and REDD1 in cells (Feng et al., 2005). Therefore, reduction in p53 may in part contribute to the up-regulation of the Akt-mTOR signaling pathway, which promotes the translation of survivin in the HDAC2 up-regulated MCF7-TamC3 cells.

**FIGURE 7:**
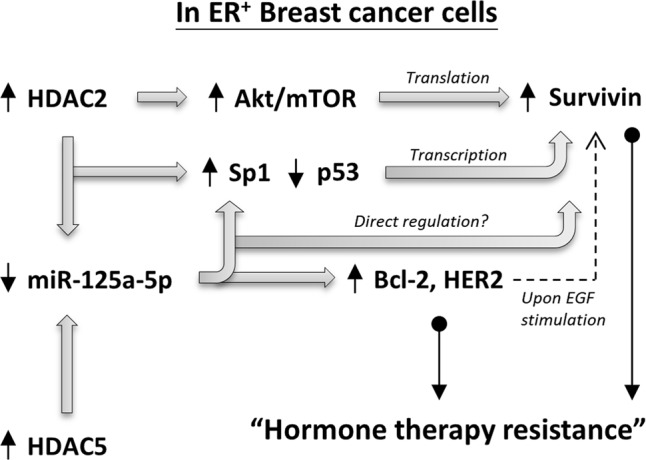
Schematic diagram showing the HDAC2/HDAC5 regulating molecular network in ER^+^ breast cancer cells.

The microRNA 125a-5p (miR-125a-5p) is one of the recently discovered tumor suppressors (Xu et al., 2014; Tong et al., 2015; Yin et al., 2015; Coppola et al., 2017). Hsieh et al. (2015) demonstrated that pharmacological inhibition of HDAC5 increased the expression of miR-125a-5p and promoted the induction of apoptosis in ER^+^ breast cancer cells, suggesting that proper regulation of the HDAC5-miR-125a-5p signaling pathway plays an important role in maintaining ER^+^ breast cancer cells survival. However, the role of miR-125a-5p in modulating the efficiency of tamoxifen or aromatase inhibitors in ER^+^ breast cancer cells is unclear. In addition, the regulatory roles of both HDAC2 on miR-125a-5p expression and miR-125a-5p on survivin expression have seldom been described in the past. In this study, we found that MCF7-TamC3 cells exhibit decreased expression of miR-125a-5p and increased expression of its downstream negatively regulating molecules, Bcl-2 and HER2, as compare to MCF7 cells. We also found that miR-125a-5p negatively regulates the expression of Sp1 and survivin in ER^+^ breast cancer cells. Over-expression of the EGF receptor, HER2, is known to be associated with tamoxifen resistance in human breast cancer cells. Shou et al. (2004) previously demonstrated that tamoxifen behaved as an estrogen agonist in their engineered ER^+^ tamoxifen-resistant MCF-7/HER-2 breast cancer cells that expressed high levels of AIB1 and HER2. Considering that the medium used in this study for culturing MCF7 and MCF7-TamC3 cells did not contain any additional EGF, the over-expressed HER2 in MCF7-TamC3 may not be a major cause for the induction of estrogen-independency and tamoxifen resistance in MCF7-TamC3 cells. However, ER^+^ breast cancer cells are known to be capable of switching the hormone dependency from estrogen to EGF for their cell survival, and activation of HER2 can increase survivin expression in cancer cells. Therefore, HDAC2 and HDAC5- up-regulated HER2 expression may provide further support for survivin over-expression and tamoxifen/aromatase inhibitors resistance induction in patients with ER^+^ breast cancer cells upon EGF stimulation (**Figure 7**) (Papanikolaou et al., 2011).

Autophagy is a double-edged sword. Up-regulation of autophagy can promote the survival of cells under genotoxic stress, metabolic stress and energy starvation (Ogata et al., 2006; Qiang et al., 2013). However, prolonged autophagy may reduce cell viability by promoting autophagic death (Baehrecke, 2005). Tamoxifen is an autophagy inducer and can induce autophagic cell death in a variety of cells, including retinal photoreceptor cells, glioblastoma cells, and breast cancer cells (Bursch et al., 1996; Cho et al., 2012; Graham et al., 2016). Inhibiting autophagy by the pharmacological inhibitor, 3MA, partially prevented tamoxifen-induced cell death in MCF7 cells (Bursch et al., 1996). Noticeably, Bicaku et al. (2007) showed that the anti-tumor effects of tamoxifen in breast cancer cells were enhanced by the co-administration of HDAC inhibitors, and the synergistic interaction was probably caused by the induction of autophagy by both modalities. Bcl-2 and mTOR are well-known negative regulators of both apoptosis and autophagy while recent evidence indicate that the anti-apoptotic molecule, survivin, also plays a negative modulatory role in autophagy in cancer cells (Pattingre et al., 2005; Alers et al., 2012; Cheng et al., 2015; Vequaud et al., 2015). MCF7 is a caspase-3 deficient breast cancer cell line (**Supplementary Figure S1B**), and targeting survivin by the small molecule inhibitor YM155 has been shown to induce caspase-independent, but autophagy-dependent, DNA damage and cell death in breast cancer cells regardless of the status of caspase-3, p53, and ER (Cheng et al., 2015). In this study, we found that the endogenous autophagy level of MCF7-TamC3 was lower than that of MCF7 cells, as indicated by the reduced expression of Atg5-Atg12 conjugate and increased expression and protein stability (half-life) of p62/SQSTM1 in MCF7-TamC3 cells. The up-regulated HDAC2 and HDAC5 may promote the development of tamoxifen or hormone therapy resistance in part by lowering the endogenous autophagic level and inhibiting tamoxifen-induced autophagy through miR-125a-5p-survivin, miR-125a-5p-Bcl-2, and Akt/mTOR-survivin signaling pathways.

## Conclusion

Dysregulation of HDAC2 and HDAC5 can be found in ER^+^, estrogen-independent, tamoxifen-resistant breast cancer cells and high expression of HDAC2 correlates with poor clinical outcomes in ER^+^ tamoxifen-treated breast cancer patients. Because HDAC2 and HDAC5 positively regulate the expression of survivin and negatively regulate the expression of miR-125a-5p in ER^+^ breast cancer cells, targeting HDAC2 and HDAC5, or their downstream regulating molecules like survivin and miR-125a-5p, may be a potential strategy for overcoming resistance to hormone therapy in patients with ER^+^ breast cancer.

## Author Contributions

W-TH, Y-HT, S-HC, C-WK, and CHAC conceived and designed the experiments. W-TH, Y-HT, C-WK, PCW, SMC, and C-HL performed the experiments. Y-HT, C-WK, Y-LK, K-TL, W-CC, C-YC, and Y-CC analyzed the data. EYL and CHAC wrote and proofread the paper.

## Conflict of Interest Statement

The authors declare that the research was conducted in the absence of any commercial or financial relationships that could be construed as a potential conflict of interest.

## Footnotes

1 http://kmplot.com/analysis/

2 http://xvm145.jefferson.edu/progmir/

3 http://www.targetscan.org/vert_71/

4 http://pictar.mdc-berlin.de/

## Supplementary Material

FIGURE S1 Molecular characteristics of human breast MCF7, MCF7-TamC3, and ZR-75-1 cancer cells. **(A,B)** Expression of ER-α and caspase-3 in different breast cancer cells was determined by the Western blot analysis.

FIGURE S2 mTOR regulates the expression of survivin in breast cancer cells. MCF7, MCF7-TamC3, and ZR-75-1 cells were treated with the mTOR inhibitor, rapamycin, for 48 h and expression of various proteins was determined by the Western blot analysis.

FIGURE S3 HDAC2 regulates p53 and Sp1 expression in ER^+^ breast cancer cells. **(A)** Results of the protein–protein interaction (PPI) networks analysis generated by the web-based software STRING version 10.0 (http://string-db.org/) showing possible regulations of survivin (*BIRC5*) expression *via* p53 (*TP53*) and Sp1 (*SP1*)-dependent mechanisms. Minimum required interaction score was set to 0.700 (high confidence) for the analysis. **(B)** MCF7 cells were transfected with scramble siRNA, HDAC2 siRNA, or HDAC2 siRNA together with the p53 inhibitor, pifithrin-α, for 24 h. Expression of survivin was determined by the Western blot analysis.

FIGURE S4 HDAC5 positively modulates Sp1 expression in ER^+^ breast cancer cells. **(A)** The Sp1/miR-125a-5p interaction was predicted using miRNA target prediction software TargetScan (http://www.targetscan.org/vert_71/) and PicTar (http://pictar.mdc-berlin.de/). **(B)** MCF7-TamC3 and ZR-75-1 cells were transfected with either scramble or HDAC5 siRNA for 24 h and expression of Sp1 was determined by Western blotting.

## Abbreviations

EGF: epidermal growth factor
ER^+^: estrogen receptor positive
HDAC: histone deacetylase
HER2: human epidermal growth factor receptor 2
ROC: receiver operating characteristic curve
