# Supplementary figures and images for: HDAC2 and HDAC5 Up-Regulations Modulate Survivin and miR-125a-5p Expressions and Promote Hormone Therapy Resistance in Estrogen Receptor Positive Breast Cancer Cells
Source: Front Pharmacol. 2017 Dec 13;8:902. doi: 10.3389/fphar.2017.00902 (PMC5736991; doi:10.3389/fphar.2017.00902)

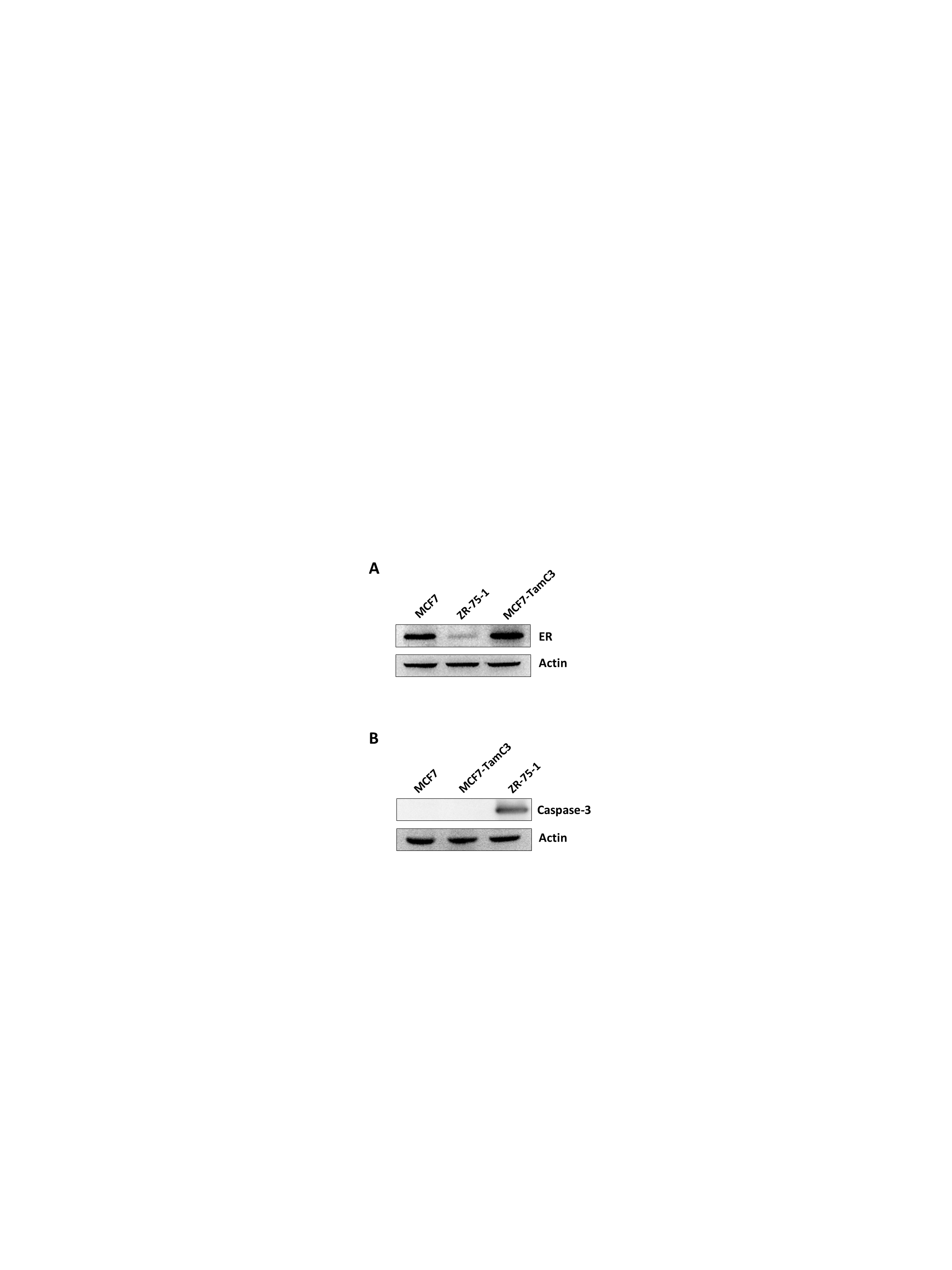

Supplement: FIGURE S1 — Molecular characteristics of human breast MCF7, MCF7-TamC3, and ZR-75-1 cancer cells. (A,B) Expression of ER-α and caspase-3 in different breast cancer cells was determined by the Western blot analysis. [file Image_1.TIF]

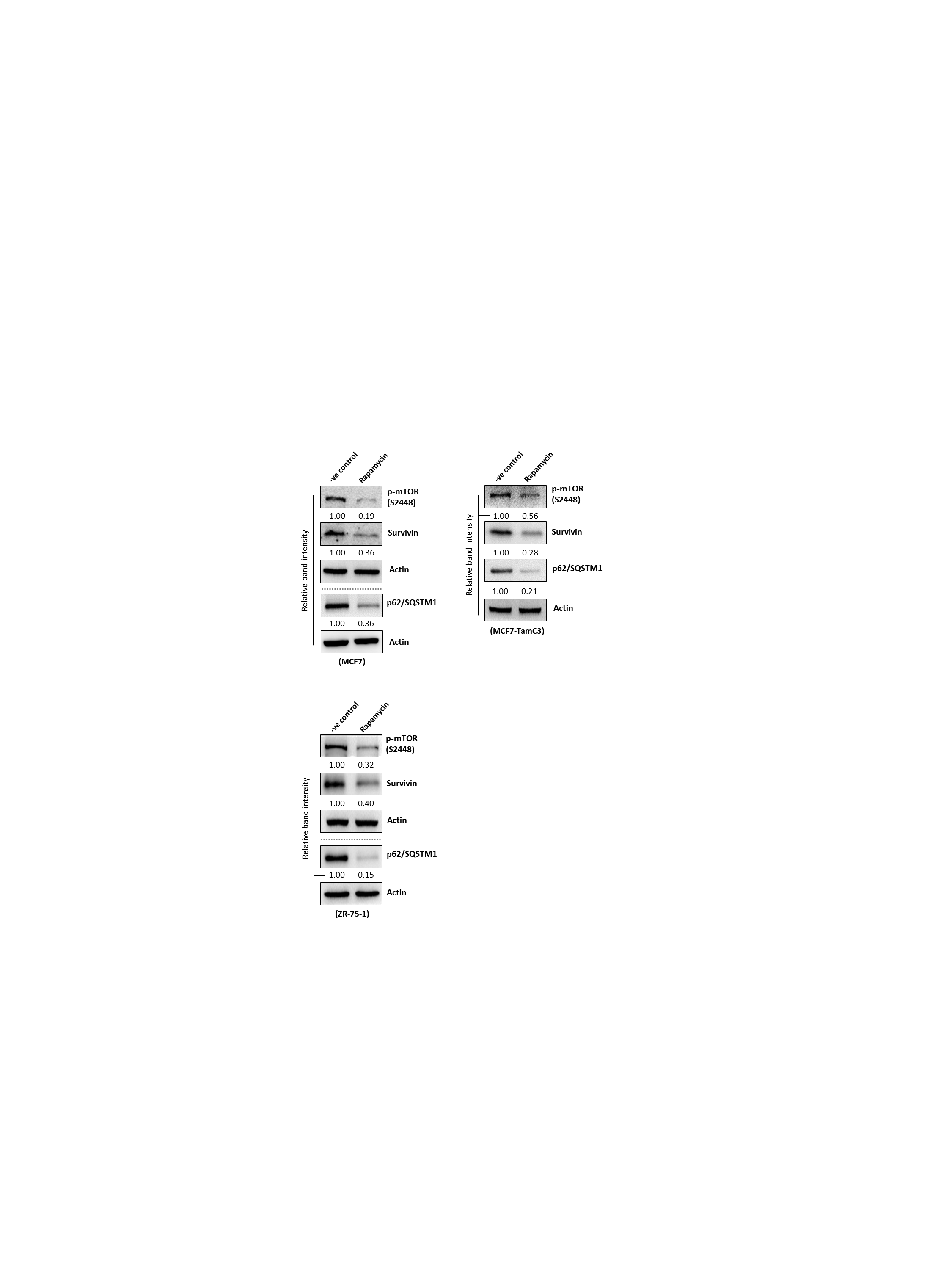

Supplement: FIGURE S2 — mTOR regulates the expression of survivin in breast cancer cells. MCF7, MCF7-TamC3, and ZR-75-1 cells were treated with the mTOR inhibitor, rapamycin, for 48 h and expression of various proteins was determined by the Western blot analysis. [file Image_2.TIF]

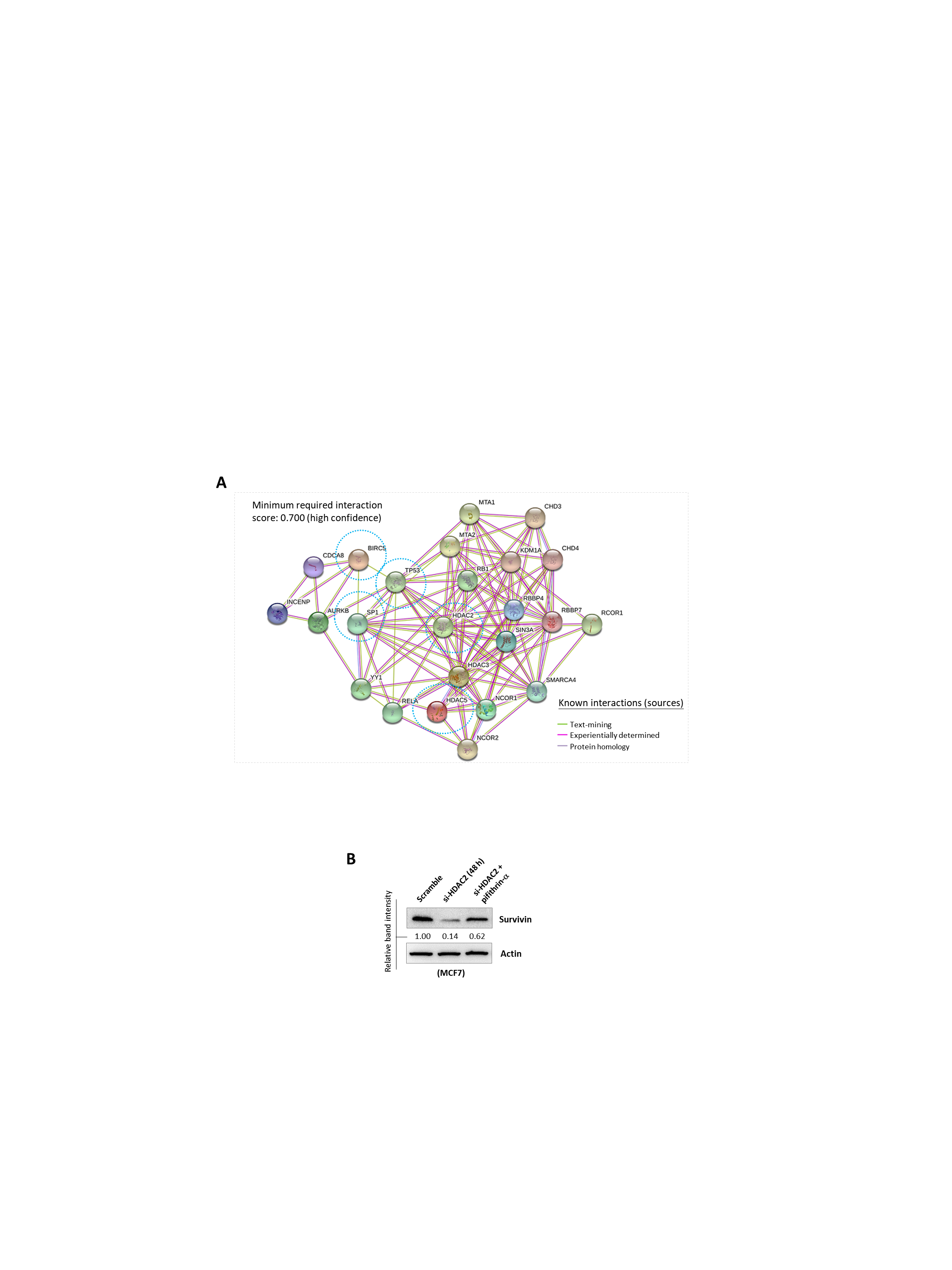

Supplement: FIGURE S3 — HDAC2 regulates p53 and Sp1 expression in ER+ breast cancer cells. (A) Results of the protein–protein interaction (PPI) networks analysis generated by the web-based software STRING version 10.0 (http://string-db.org/) showing possible regulations of survivin (BIRC5) expression via p53 (TP53) and Sp1 (SP1)-dependent mechanisms. Minimum required interaction score was set to 0.700 (high confidence) for the analysis. (B) MCF7 cells were transfected with scramble siRNA, HDAC2 siRNA, or HDAC2 siRNA together with the p53 inhibitor, pifithrin-α, for 24 h. Expression of survivin was determined by the Western blot analysis. [file Image_3.TIF]

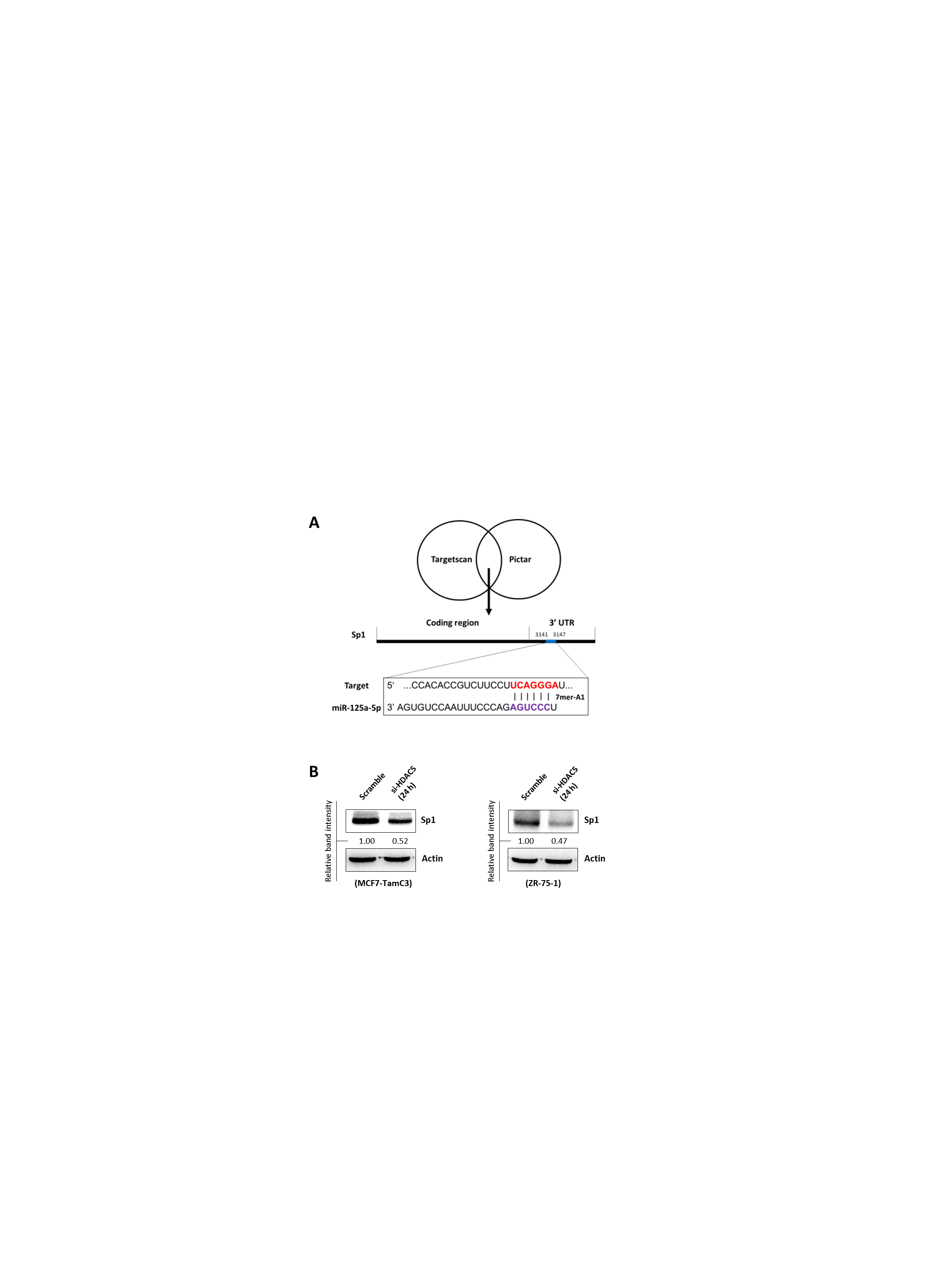

Supplement: FIGURE S4 — HDAC5 positively modulates Sp1 expression in ER+ breast cancer cells. (A) The Sp1/miR-125a-5p interaction was predicted using miRNA target prediction software TargetScan (http://www.targetscan.org/vert_71/) and PicTar (http://pictar.mdc-berlin.de/). (B) MCF7-TamC3 and ZR-75-1 cells were transfected with either scramble or HDAC5 siRNA for 24 h and expression of Sp1 was determined by Western blotting. [file Image_4.TIF]
